# Silicon-Cantilever-Enhanced Single-Fiber Photoacoustic Acetylene Gas Sensor

**DOI:** 10.3390/s23177644

**Published:** 2023-09-03

**Authors:** Zhengyuan Zhang, Xinhong Fan, Yufu Xu, Yongqi Wang, Yiyao Tang, Rui Zhao, Chenxi Li, Heng Wang, Ke Chen

**Affiliations:** 1 State Grid Gansu Electric Power Research Institute, Lanzhou 730030, China; zzy3377512@foxmail.com (Z.Z.); shinhung@163.com (X.F.); wangyongqi@126.com (Y.W.); 15101206230@163.com (Y.T.); 18909408257@163.com (R.Z.); 2 School of Optoelectronic Engineering and Instrumentation Science, Dalian University of Technology, Dalian 116024, China; lichenxi@mail.dlut.edu.cn (C.L.); wangheng190614@163.com (H.W.)

**Keywords:** photoacoustic spectroscopy, single-fiber photoacoustic sensor, trace acetylene gas analysis, white-light interference demodulation

## Abstract

A single-fiber photoacoustic (PA) sensor with a silicon cantilever beam for trace acetylene (C_2_H_2_) gas analysis was proposed. The miniature gas sensor mainly consisted of a microcantilever and a non-resonant PA cell for the real-time detection of acetylene gas. The gas diffused into the photoacoustic cell through the silicon cantilever beam gap. The volume of the PA cell in the sensor was about 14 μL. By using a 1 × 2 fiber optical coupler, a 1532.8 nm distributed feedback (DFB) laser and a white light interference demodulation module were connected to the single-fiber photoacoustic sensor. A silicon cantilever was utilized to improve the performance when detecting the PA signal. To eliminate the interference of the laser-reflected light, a part of the Fabry–Perot (F-P) interference spectrum was used for phase demodulation to achieve the highly sensitive detection of acetylene gas. The minimum detection limit (MDL) achieved was 0.2 ppm with 100 s averaging time. In addition, the calculated normalized noise equivalent absorption (NNEA) coefficient was 4.4 × 10^−9^ W·cm^−1^·Hz^−1/2^. The single-fiber photoacoustic sensor designed has great application prospects in the early warning of transformer faults.

## 1. Introduction

With the increasing demand for electricity in various industries, the power system is developing in the directions of large capacity, ultra-high voltage and intelligence. The continuous online monitoring of electrical equipment can grasp the internal insulation status of transformer equipment in a timely manner and find hidden accidents in equipment operation, so as to prevent the development of transformer latent faults as early as possible [1,2]. The online monitoring of dissolved acetylene (C_2_H_2_) gas analysis in transformer oil is meaningful for transformer fault diagnosis [3]. Currently, dissolved gas analysis (DGA) is considered to be the most universal method. The concentration of acetylene gas dissolved in transformer oil can reflect the severity of the discharge fault in oil-immersed electrical equipment [4,5]. Consequently, high-sensitivity acetylene detection is often required for the early warning of transformer faults. Photoacoustic (PA) spectroscopy (PAS) enables the realization of online monitoring of dissolved gas in transformer oil, with advantages of high sensitivity, no background detection and anti-electromagnetic interference. Overheating and discharge faults in oil-immersed transformers can be distinguished by detecting acetylene gas [6,7,8,9,10,11,12].

The common methods for measuring dissolved gas in transformer oil mainly include gas chromatography, Raman spectroscopy and tunable diode laser spectroscopy technology (TDLAS). Gas chromatography is a method of separating different gases using chromatographic columns for the quantitative detection of multicomponent gas [13]. However, the gas chromatograph requires frequent calibration and maintenance, and the operation is cumbersome. Tunable diode laser spectroscopy technology can calculate the concentration of gas by detecting the change in transmission light intensity. However, TDLAS is easily affected by the power influence of the light source, and the actual detection accuracy is low [14]. Compared with these dissolved gas analysis methods, PAS has the advantages of no carrier gas, no frequent calibration, a small gas sample amount and high sensitivity, which is gradually replacing traditional dissolved gas analysis methods and has received widespread attention in recent years [15,16,17,18,19,20,21]. A different resonance principle and photoacoustic cells with different shapes were proposed. These included H-type resonant photoacoustic cells, T-type resonant photoacoustic cells, Helmholtz resonant photoacoustic cells and multi-pass absorption cells [22,23,24,25,26,27]. The sound pressure generated by gas absorption was greatly amplified by the resonant photoacoustic cell and multi-pass absorption cell. With the development of laser and acoustic sensors, distinct photoacoustic systems for trace gas analysis have appeared, including traditional photoacoustic spectroscopy based on a microphone [28,29], quartz-enhanced photoacoustic spectroscopy (QEPAS) [30,31], and cantilever-enhanced photoacoustic spectroscopy (CEPAS) [32].

The traditional photoacoustic spectroscopy detection systems were mainly composed of a light source, photoacoustic cell and acoustic sensor. The volume was relatively large. In recent years, a small-volume fiberoptic photoacoustic sensing probe has been studied. So as to overcome the common problems of traditional photoacoustic spectroscopy detection systems, such as weak anti-electromagnetic interference ability and a large gas consumption chamber, a miniaturized fiber optic sensor has gradually attracted the interest of researchers. The photoacoustic sensor integrated a photoacoustic cell and a Fabry–Perot (F-P) interference cavity. At present, a dual-fiber photoacoustic sensor has been proposed to detect dissolved gas in oil. Chen et at. proposed a high-sensitivity fiberoptic photoacoustic sensor for the in situ detection of dissolved gases in oil [33]. The sensor included a micro photoacoustic cell and an acoustic sensitive element. The two air chambers are connected, and the volume of the photoacoustic cell is 70 μL. However, the optical fiber sensor needs to be connected to two optical fibers, one of which excites light for gas absorption to produce photoacoustic signals, and the other of which transmits probe light for the oscillation amplitude of acoustic sensitive elements. Moreover, the sensor has the problems such as the fiber resources is occupied more and it being difficult to further reduce the volume of the photoacoustic cell. The photoacoustic signal is obtained by using the white light interference demodulation module [34]. The detection limit of dissolved acetylene gas reached 0.5 μL/L. A stable and high-speed F-P demodulation module is the soul of the acoustic sensing element in the fiber optic photoacoustic sensing system. Phase demodulation is a highly stable demodulation method [35]. Zhou et al. integrated the miniature photoacoustic cell with the Fabry–Perot (F-P) acoustic sensor [36], and realized the in situ all-optical signal detection of dissolved gas in transformer oil through the external oil–gas separation film. The two ends of the photoacoustic sensor are connected to a fiber for transmitting the excitation light and the detection light, respectively. These sensors required two optical fibers to enter the air chamber, making it difficult to further reduce the volume. Microscopic gas chambers utilizing hollow-core optical fibers have recently been shown to be useful for trace gas detection. Jin et al. proposed a photothermal gas sensor with a hollow photonic bandgap optical fiber F-P absorption cell [37]. The detection light and the excitation light were transmitted in the same hollow photonic bandgap optical fiber, and the light and gas had better absorption efficiency. In order to reduce the consumption and response time of the detection method of dissolved acetylene in oil, a small-volume T-type photoacoustic cell was proposed and the sampling oil amount of 50 mL was detected via headspace degassing. The detection limit of dissolved acetylene was 0.2 μL/L [38]. In order to reduce the volume of the fiber optic photoacoustic sensor probe and maximize the integration, Li et al. proposed a single-fiber photoacoustic sensor for the detection of trace methane gas. The minimum detectable limit (MDL) was 8.4 ppm, with a 1 s lock-in integral time [39]. However, the fiber optic photoacoustic sensor system was limited by the channel band of the wavelength division multiplexer and it was difficult to detect other gases, such as acetylene gas. Moreover, how the demodulation module eliminated the interference spectrum to achieve a stable, high signal-to-noise ratio demodulation was not considered.

In this paper, we propose a single-fiber photoacoustic (PA) sensor for the detection of trace acetylene gas without gas valves and pumps. The single fiber photoacoustic sensor integrated a photoacoustic cell and an F-P interference cavity. By using a 1 × 2 fiber optical coupler, a 1532.8 nm distributed feedback (DFB) laser and a white light interference demodulation module were coupled in the single-fiber photoacoustic sensor. The single-fiber photoacoustic sensor used the silicon cantilever as an acoustic sensitive element to improve detection sensitivity. At the same time, the diameter and length of the cylindrical PA cell were optimized to weaken the influence of the small volume cavity on the silicon cantilever beam and facilitate the mechanical polishing of the inner wall of the photoacoustic cell. The sensor was miniaturized and intrinsically safe, with a diffusion gap. The influence of PA cell length on the detection performance of single fiber photoacoustic sensor was analyzed theoretically. The volume of the PA cell in the single-fiber photoacoustic sensor was about 14 μL. A silicon cantilever was used to detect the sound pressure. The second-harmonic wavelength modulation spectroscopy (2*f*-WMS) method was used to measure PA signals. The single-fiber photoacoustic sensor designed has great application prospects in the early warning of transformer faults.

## 2. Design of the Sensing System

### 2.1. Design of the Single-Fiber Photoacoustic Sensor

**Figure 1:**
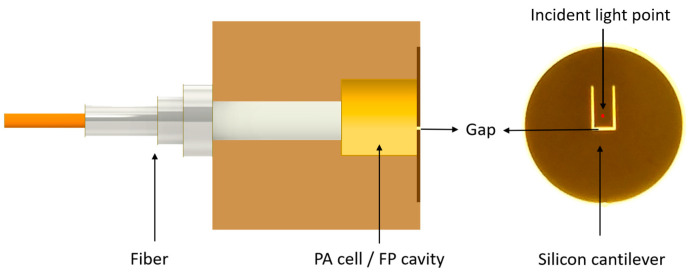
Schematic structure of the single-fiber photoacoustic sensor.

Figure 1 shows the schematic structure of the single-fiber photoacoustic (PA) sensor, in which a single-mode fiber transmits excitation light and detection light at the same time by using a 1 × 2 fiber optical coupler. The single-fiber photoacoustic sensor integrated together a PA cell and an F-P cavity. The main component of the single-fiber photoacoustic sensor was the silicon cantilever beam. The silicon cantilever beam was fixed at the end of the cylindrical photoacoustic cell. The output light of the optical fiber was vertically aligned 0.2 mm above the free end of the cantilever beam. The PA cell and the F-P cavity had the same cylindrical cavity. The diameter of the PA cell was 3 mm. The radius of the tube was suitable for polishing the inner wall of the tube to reduce the background signal generated by the wall absorption. The outer diameter of the single-fiber photoacoustic sensor was 8 mm, slightly larger than the diameter of the silicon cantilever beam. The length of the silicon cantilever was 1.6 mm, and the air gap size was 6 μm. Gas can diffuse into the PA cell through the gap in the silicon cantilever beam.

For a non-resonant photoacoustic cell, the sound pressure generated internally is uniform and the value can be expressed as [40]:

(1) $$P=\frac{CP_{0}\alpha (v)(\gamma -1)}{\pi R_{c}^{2}w}\frac{1}{\sqrt{1+{\left(\frac{1}{w\tau_{c}}\right)}^{2}}}$$

where *P*_0_ is the effective optical power, α(ν) is the absorption coefficient of the gas at the wavenumber ν, γ is the specific heat capacity of the gas, Rc is the radius of the cylindrical photoacoustic cell, *w* represents the modulation frequency and *C* represents the concentration of the gas to be measured. When the photoacoustic spectroscopy measurement system has been determined, the intensity of the photoacoustic signal changes linearly with the gas concentration. For non-resonant photoacoustic cells, the smaller the volume of the PA cavity, the stronger the photoacoustic signal.

The interferometric structure of the single-fiber photoacoustic sensor was an extrinsic Fabry–Perot interferometer (EFPI), whose demodulation resolution could be determined by the visibility of the fringe pattern. The coupling coefficient can be expressed as [41]:

(2) $$\varepsilon (2l)=\frac{2r(2l)r_{0}}{r_{0}^{2}+r^{2}(2l)}$$

where l is the F-P cavity length and *r*_0_ is the mode-field radius in the fiber. The numerical aperture (NA) is 0.14 and the mode-field radius *r*_0_ is calculated to be 4.61 μm. The mode-field radius of the reflective optical filed r(2d) is

(3) $$r(2d)=r_{0}\sqrt{1+{\left(\frac{2d\lambda }{\pi r_{0}^{2}}\right)}^{2}}$$

The visibility *v* of the EFPI can be expressed as

(4) $$v=\frac{I_{MAX}-I_{M\mathrm{IN}}}{I_{MAX}+I_{M\mathrm{IN}}}=\frac{2(1-R_{1})\varepsilon (2l)\sqrt{R_{1}R_{2}}}{R_{1}+{\left(1-R_{1}\right)}^{2}R_{2}\varepsilon^{2}(2l)}$$

where *I_MAX_* and *I_MIN_* are the maximum and the minimum of the light intensity, respectively. *R*_1_ and *R*_2_ are the reflectivity of the fiber tip and the cantilever, respectively. *R*_1_ of the SMF28 single-mode fiber is ~4% and *R*_2_ of the reflection surface of cantilever beam is ~90%. The light intensity of the two-beam interference is

(5) $$I_{R}=I_{0}(R_{1}+{\left(1-R_{1}\right)}^{2}R_{2}\varepsilon^{2}(2l))(1+v\cos(\frac{4\pi l}{\lambda }+\pi ))$$

**Figure 2:**
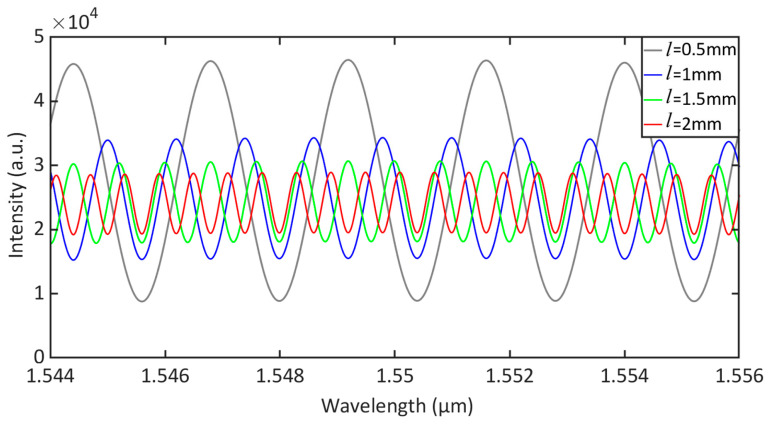
Simulated interference spectrum of F-P cavity at different cavity lengths.

The silicon cantilever beam had pressed vibration from the sine pressure generated by the acetylene gas absorption. The white light interferometric demodulation module processed the small movement of the interference spectrum to obtain the change in F-P cavity length; that is, the photoacoustic signal was detected via the silicon cantilever beam. The amounts of interference spectral fringe cycles and contrast under different cavity lengths were analyzed. High-quality interference fringes can ensure the stability of the demodulation module. According to Formula (5), *I*_0_ was approximately set as a function of the Gaussian distribution of the output light intensity with the wavelength, and the wavelength range was set to 1544 nm–1556 nm. The interference light intensities under 0.5 mm, 1 mm, 1.5 mm and 2 mm cavity lengths were simulated using MATLAB R2018a. Figure 2 shows the interference spectrum of the F-P cavity at different cavity lengths. While the F-P cavity length increased from 0.5 mm to 2 mm, the number of interference spectrum periods increased linearly. Due to the sampling point of the white light interference, the demodulation module was certain. Consequently, the number of interference spectrum periods should be within the demodulation range. The intensity of interference spectrum increased nonlinearly with the increase in F-P cavity length. To increase the signal of the non-resonant photoacoustic cell as much as possible, the length of the F-P cavity was set to 2 mm.

### 2.2. Experimental Setup

**Figure 3:**
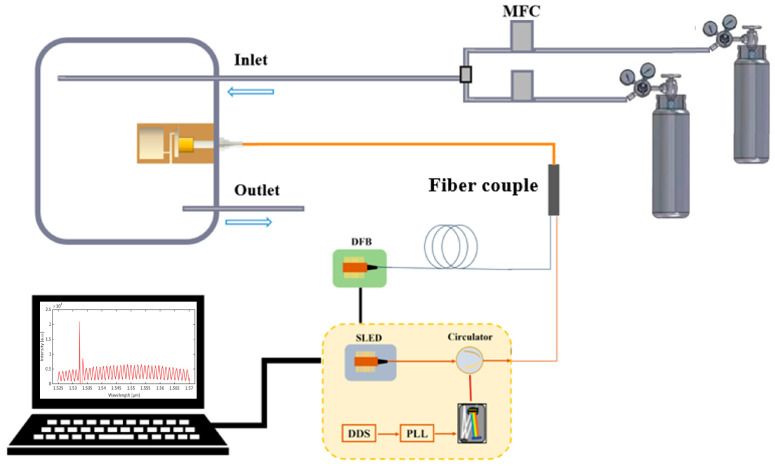
Schematic structure of the test system for the single-fiber photoacoustic trace acetylene sensor; MFC: Mass-Flow Controller.

Figure 3 depicts the schematic structure of the single-fiber photoacoustic acetylene gas sensing system. A tunable distributed feedback (DFB) laser was used as an excitation laser to pump acetylene gas molecules. The DFB laser was driven by the current of sinusoidal and triangular waves. By using the 1 × 2 fiber-optical coupler, the distributed feedback (DFB) laser and the white light interference demodulation module were coupled in the single-fiber photoacoustic sensor. The length of the detection distance depended on the length of the single fiber. Consequently, the single fiber photoacoustic sensing system could be used for long-distance detection. The absorption line of acetylene gas was scanned by changing the output wavelength of the DFB laser. The second harmonic signal generated by acetylene gas absorption was obtained using the white light interference demodulation module. A super-luminescent light-emitting diode (SLED) was used as a broad-spectrum probe source. The two beams through the end face of the fiber and the reflective surface of the cantilever caused interference. Since the wavelength of the excitation light was near the center wavelength of the SLED, the two beams created crosstalk. Two mass-flow controllers (MFC) were used to prepare gases of different concentrations. The single-fiber photoacoustic sensor was placed in the gas chamber to detect the concentration of acetylene. The excitation light and detection light were coupled to the same fiber by the fiber coupler. The high-speed white light interference demodulation method was used to detect the reflected probe light generated by F-P interference. It was then processed with a field programmable gate array (FPGA)-based lock-in amplifier.

**Figure 4:**
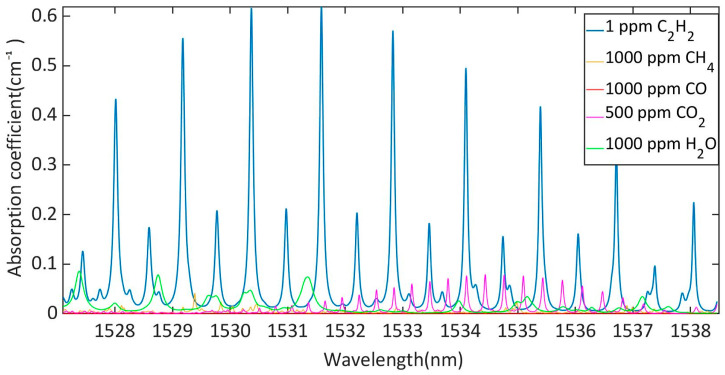
The absorption coefficients of 1 ppm acetylene, 1000 ppm methane, 1000 ppm carbon monoxide, 1000 ppm water and 500 ppm carbon dioxide at 1527.1 nm to 1538.5 nm, plotted according to HITRAN data.

According to the HITRAN database, gas absorption lines of acetylene, water and carbon dioxide were obtained. As can be seen from Figure 4, there was 1 ppm acetylene, 1000 ppm methane, 1000 ppm carbon monoxide, 1000 ppm water and 500 ppm carbon dioxide interference at 1527.1 nm to 1538.5 nm. Acetylene had a large absorption coefficient at 1532.8 nm and 1531.6 nm. Considering the interference of H_2_O and CO_2_ in the air, 1532.83 nm DFB laser was selected as the detection wavelength of acetylene. Moreover, near the center of the absorption line at 1532.8 nm, acetylene was almost unaffected by CH_4_ and CO. Therefore, a DFB laser with a central wavelength of 1532.8 nm was selected as the excitation light source. However, white light interferometer interrogators have a wavelength range of 1525 nm–1570 nm. As a result, the excitation light is reflected by the photoacoustic sensor and is superimposed into the F-P interference spectrum.

**Figure 5:**
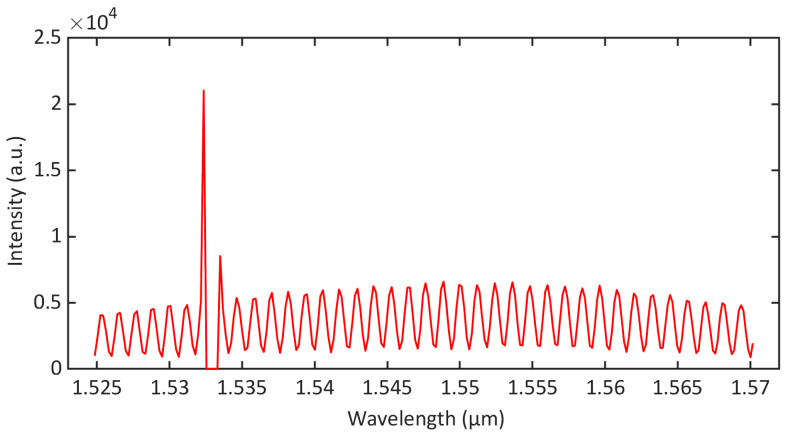
Measured F-P interferometric spectrum interfered by excitation light with a central wavelength of 1532.8 nm.

The 1532.8 nm distributed feedback (DFB) laser and the white light interference demodulation module were connected in the single-fiber photoacoustic sensor with the 1 × 2 fiber optical coupler. The F-P interferometric spectrum interfered by excitation light with a central wavelength of 1532.8 nm was obtained, as shown in Figure 5. The full interference spectrum of the F-P cavity was affected by the excitation light reflected to the fiber at the wavelengths around 1532.8 nm. The power of the DFB laser was 19.1 mW, which was greater than the power of the SLED. In order to avoid demodulation errors such as mode hopping, the feasible method in this article was to cut off the disturbed interference spectrum. The F-P cavity length was phase-demodulated using the interference spectrum in the wavelength range of 1535–1570 nm to enable the highly sensitive detection of the photoacoustic signal.

## 3. Experimental Results

### 3.1. Frequency Response

**Figure 6:**
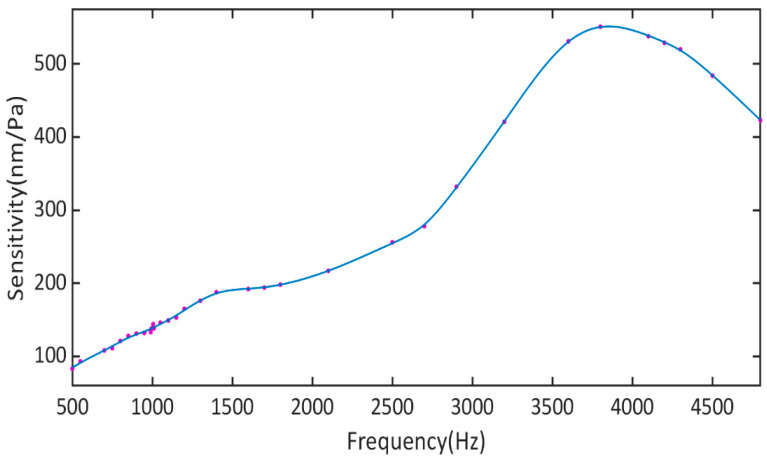
Amplitude-frequency response of the single-fiber photoacoustic sensor.

By testing the sensitivity of the cantilever beam to the sound pressure, the detection performance of the single-fiber photoacoustic sensor could be reflected to some extent. An acoustic test system was established to assess the sound pressure sensitivity of the single-fiber photoacoustic sensor. The static cavity length of the F-P cavity was measured to be 2049.16 nm; meanwhile, the volume of the PA cavity was 14 μL. Figure 6 shows the amplitude–frequency response of the single-fiber photoacoustic sensor. The response of the single-fiber photoacoustic sensor to sound pressure was stable near 1000 Hz, with a strong anti-interference ability to the environment. When the frequency of the silicon cantilever beam was set to 1000 Hz, the sensitivity was 139.5 nm/Pa. The resonant frequency of the cantilever beam was 4000 Hz. However, with the small F-P cavity, the cantilever beam was affected by the air dumping. The frequency response curve of the cantilever beam drifted easily due to the change in the ambient temperature in the single fiber photoacoustic sensor. Consequently, the working frequency of the single-fiber photoacoustic sensor was selected to be far away from the resonant frequency of the silicon cantilever beam. According to Formula 1, for the non-resonant photoacoustic cell, the size of the photoacoustic signal decreased as the operating frequency increased. The sensitivity fluctuation of the silicon cantilever beam at 1000 Hz was relatively small. While the sensitivity of the silicon cantilever beam was relatively small, it was less affected by environmental noise. Moreover, the photoacoustic signal generated by the non-resonant photoacoustic cell at 1000 Hz was relatively large.

**Figure 7:**
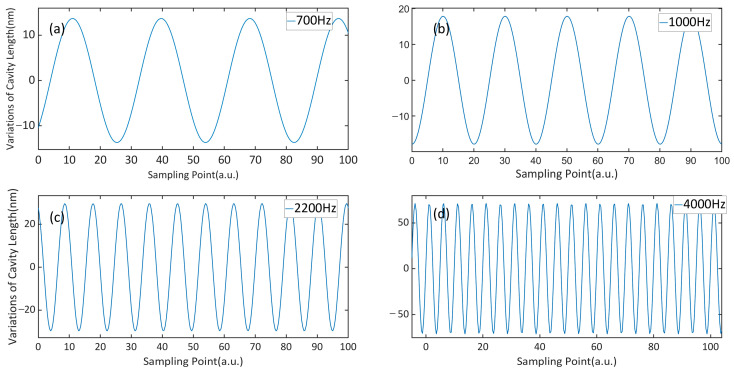
Time domain response at the frequency of (**a**) 700 Hz, (**b**) 1000 Hz, (**c**) 2200 Hz and (**d**) 4000 Hz with the sound pressure of 120 mPa.

The sound pressure on a reference microphone (4189, B&K) and the silicon cantilever was adjusted to 120 mPa and the time domain responses of the cantilever were, respectively, measured at the frequencies of 700 Hz, 1000 Hz, 2200 Hz and 4000 Hz, as shown in Figure 7. The resonant frequency of the cantilever beam was around 4000 Hz. The time domain signal of the single-fiber photoacoustic sensor became denser with the increase in frequency. The variations in cavity length were detected by the white light interference demodulation module. While the sound pressure was 120 mPa, the displacement of the silicon cantilever beam swinging up and down was about 36 nm at 1000 Hz frequency.

### 3.2. Concentration Measurement

**Figure 8:**
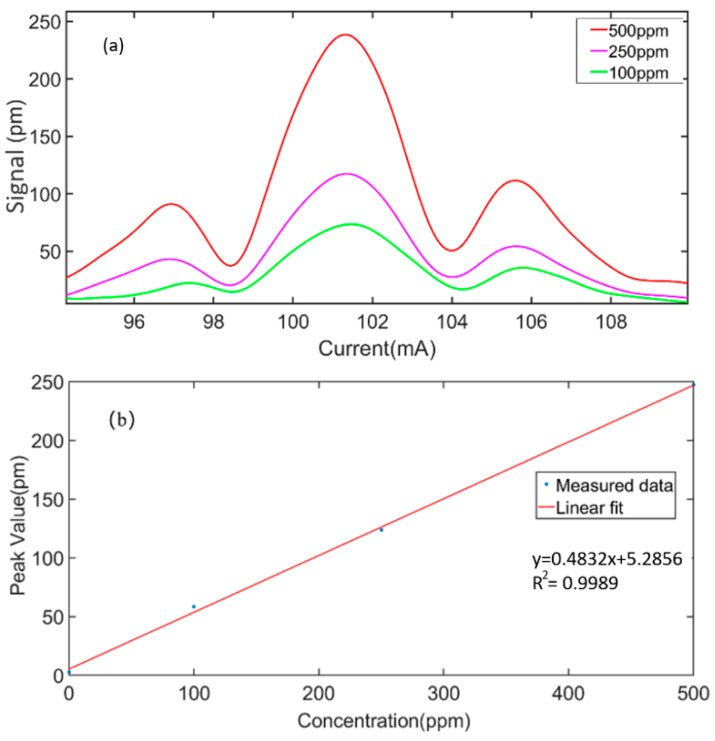
(**a**) Second-harmonic signals of 100 ppm, 250 ppm and 500 ppm acetylene were measured. (**b**) Peak of the second-harmonic photoacoustic signal as a function of C_2_H_2_ concentration.

The PA signal of the single-fiber photoacoustic sensing system was measured with different concentrations of C_2_H_2_ gas. The bias current of the DFB laser was increased from 95 mA to 110 mA. C_2_H_2_/N_2_ gas mixtures of 100 ppm, 250 ppm and 500 ppm were, respectively, diffused into the PA cell of the single-fiber photoacoustic senor. The modulation frequency of the DFB laser was set to 500 Hz, and the generated second harmonic signal was detected. Consequently, the detection frequency of the cantilever beam was 1000 Hz. The root mean square (RMS) value of the second harmonic signal was measured by the lock-in amplifier in the FPGA. Figure 8a shows the second harmonic signal of the single-fiber photoacoustic sensor at the concentrations of 100 ppm, 250 ppm and 500 ppm, respectively. While the wavelength and bias current of the DFB laser were 1532.8 nm and 4.5 mA, respectively, which corresponds to the gas absorption peak, the second harmonic PA signal value was the largest. Figure 8b shows the peak of the second-harmonic photoacoustic signal as a function of C_2_H_2_ concentration. The results show that the sensitivity of the single-fiber photoacoustic sensor sensing system to C_2_H_2_ gas was 0.48 pm/ppm and the regression coefficient was 0.9989, indicating that the detection system had good linearity in the concentration range of 500 ppm C_2_H_2_.

### 3.3. Detection Limit

**Figure 9:**
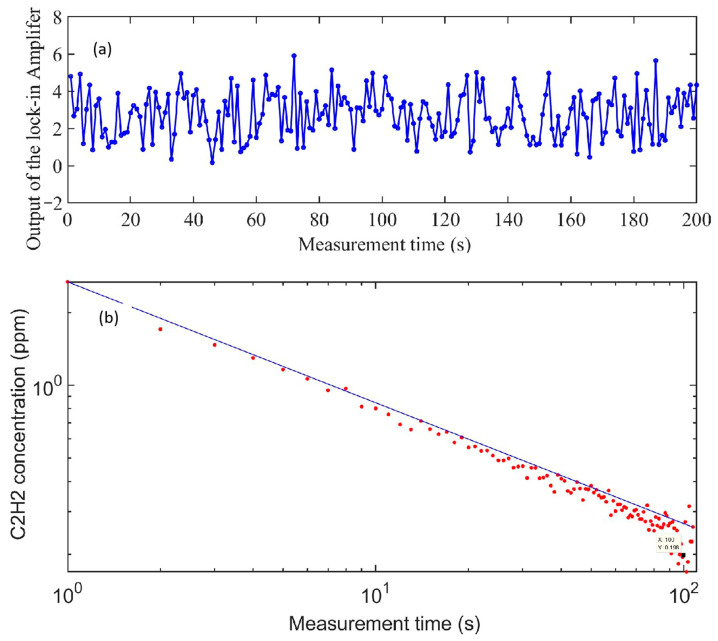
(**a**) Measured noise with the chamber filled with pure nitrogen. (**b**) Analysis results of Allan–Werle variance.

To detect the MDL of the single-fiber photoacoustic sensor, a long-term stability monitoring experiment of the single-fiber PA gas sensing system was carried out. The bias current of the DFB laser was set to 101.2 mA with a 1 s lock-in integral time. The single-fiber photoacoustic sensor was placed in a pure N_2_ environment to detect the noise level of the system within 200 s. Figure 9a,b show the analysis results of Allan–Werle variance as a function of the averaging time. The Allan–Werle variance shows a decreasing trend with increasing acquisition time. The MDL of C_2_H_2_ gas was 0.2 ppm with an averaging time of 100 s. In addition, the power of the DFB laser was tested to be 19.1 mW at 1532.8 nm, and the calculated normalized noise equivalent absorption (NNEA) coefficient was 4.4 × 10^−9^ W·cm^−1^·Hz^−1/2^. In order to reflect the small volume and high sensitivity performance of the proposed sensor, Table 1 shows the comparison of the NNEA/MDL and the chamber volume with other miniaturized photoacoustic sensors.

## 4. Conclusions

In conclusion, a single-fiber photoacoustic (PA) sensor has been proposed for trace acetylene (C_2_H_2_) gas analysis with the advantages of having high sensitivity, miniaturization and long-distance detection and of being intrinsically safe. The single-fiber photoacoustic sensor mainly consisted of a microcantilever and a non-resonant photoacoustic cell for the real-time detection of trace acetylene gas. The trace acetylene gas could diffuse into the silicon cantilever gap opened in the wall of the PA cell. The volume of the PA cell in the sensor was about 14 μL. The influence of different lengths of photoacoustic cells on the single-fiber photoacoustic sensing system was compared theoretically. The optimized length of the PA cell was 2 mm. The amplitude-frequency response of the silicon cantilever with a small inner cavity was tested and analyzed. The single-fiber photoacoustic sensor had a good linearity response for C_2_H_2_ concentration, of less than 500 ppm with a responsivity of 0.48 pm/ppm. In addition, the minimum detectable limit and the minimum detectable absorption coefficient of the single-fiber photoacoustic sensor were achieved to be 0.2 ppm and 1.2 × 10^−7^ cm^−1^ with a 100 s averaging time, respectively. The calculated NNEA coefficient was 4.4 × 10^−9^ W·cm^−1^·Hz^−1/2^. The photoacoustic sensor designed had the advantages of small volume, simple structure, high sensitivity and no need for gas valves or pumps. By changing the laser source, it is possible to detect various trace diffused gases such as CH_4_, CO_2_, NH_3_, H_2_S and C_2_H_4_.

## Figures and Tables

**Table 1:** Performance of the proposed sensor in comparison with other miniaturized photoacoustic sensors.

Scheme	NNEA/MDL	Gas Chamber Volume
Zhang in 2023 [27]	0.49 ppm	~1.57 mL
Guo in 2022 [32]	2.1 × 10^−8^ W·cm^−1^·Hz^−1/2^	31.8 μL
Chen in 2021 [33]	0.5 μL/L	74 μL
Li in 2022 [39]	2.1 × 10^−8^ W·cm^−1^·Hz^−1/2^	1.7 μL
This paper	4.4×10^−9^ W·cm^−1^·Hz^−1/2^	14 μL

## Data Availability

Not applicable.

## References

[1] Qin W.-Q., Ma G.-M., Zhang M., Wang Y., Jiang J., Zhou H., Wang X., Yan C. (2022). Quasi-Distributed Vibration Sensing System for Transformers Using a Phase-Sensitive OFDR. IEEE Trans. Ind. Electron..

[2] Yun Y., Chen W., Wang Y., Pan C. (2018). Photoacoustic detection of dissolved gases in transformer oil. Eur. Trans. Electr. Power.

[3] Su Q., Mi C., Lai L.L., Austin P. (2000). A fuzzy dissolved gas analysis method for the diagnosis of multiple incipient faults in a transformer. IEEE Trans. Power Syst..

[4] Duval M. (2002). A review of faults detectable by gas-in-oil analysis in transformers. IEEE Electr. Insul. Mag..

[5] Saha T.K. (2003). Review of modern diagnostic techniques for assessing insulation condition in aged transformers. IEEE Trans. Dielectr. Electr. Insul..

[6] Li C., Qi H., Zhao X., Guo M., An R., Chen K. (2022). Multi-pass absorption enhanced photoacoustic spectrometer based on combined light sources for dissolved gas analysis in oil. Opt. Lasers Eng..

[7] Zhou Q., Tang C., Zhu S., Chen W., Peng X. (2015). Detection of Dissolved Carbon Monoxide in Transformer Oil Using 1.567 μm Diode Laser-Based Photoacoustic Spectroscopy. J. Spectrosc..

[8] Wu Z., Gong Y., Yu Q. (2013). Photoacoustic spectroscopy detection and extraction of discharge feature gases in transformer oil based on 1.5μ tunable fiber laser. Infrared Phys. Technol..

[9] Mao X., Zhou X., Zhai L., Yu Q. (2014). Dissolved Gas-in-Oil Analysis in Transformers Based on Near-Infrared Photoacoustic Spectroscopy. Int. J. Thermophys..

[10] Chen W., Liu B., Zhou H., Wang Y., Wang C. (2012). Diode laser-based photoacoustic spectroscopy detection of acetylene gas and its quantitative analysis. Eur. Trans. Electr. Power.

[11] Wu H., Dong L., Zheng H., Liu X., Yin X., Ma W., Zhang L., Yin W., Jia S., Tittel F.K. (2015). Enhanced near-infrared QEPAS sensor for sub-ppm level H2S detection by means of a fiber amplified 1582nm DFB laser. Sens. Actuators B Chem..

[12] Zhang C., Qiao S., Ma Y. (2023). Highly sensitive photoacoustic acetylene detection based on differential photoacoustic cell with retro-reflection-cavity. Photoacoustics.

[13] Zha S.L., Liu K., Tan T., Wang G.S., Gao X.M. (2017). Application of photoacoustic spectroscopy in multi-component gas concentration detection. Acta Photonica Sin..

[14] Jiang J., Wang Z., Ma G., Song H., Zhang C. (2019). Direct detection of acetylene dissolved in transformer oil using spectral absorption. Optik.

[15] Chen K., Wang N., Guo M., Zhao X.Y., Qi H.C., Li C.X., Zhang G.Y., Xu L. (2023). Detection of SF6 gas decomposition component H2S based on fiber-optic photoacoustic sensing. Sens. Actuators B Chem..

[16] Qi H., Xu Y., Yang L., Zhao X., Li C., Guo M., Chen K. (2023). Detection of gaseous halocarbon refrigerants and extinguishing agent based on photoacoustic spectroscopy. Sens. Actuators B Chem..

[17] Zhang B., Jia Y.J., Zhao B.L., Zhu X.S., Shi Y.W. (2023). Highly sensitive photoacoustic gas sensor with micro-embedded acoustic resonator for gas leakage detection. Opt. Lett..

[18] Chen K., Yu Q., Gong Z., Guo M., Qu C. (2018). Ultrahigh sensitive fiber-optic Fabry-Perot cantilever enhanced resonant photoacoustic spectroscopy. Sens. Actuators B Chem..

[19] Zhang L., Jiang Y., Gao H., Jia J., Cui Y., Wang S., Hu J. (2019). Simultaneous Measurements of Temperature and Pressure with a Dual-Cavity Fabry–Perot Sensor. IEEE Photonics Technol. Lett..

[20] Zhao X., Qi H., Xu Y., Li C., Guo M., Zhao J., Cui D., Chen K. (2023). Dynamic detection of ppb-level SO2 based on differential photoacoustic cell coupled with UV-LED. Opt. Lett..

[21] Zhao X., Guo M., Cui D., Li C., Qi H., Chen K., Ma F., Huang J., Zhang G., Zhao J. (2023). Multi-pass Differential Photoacoustic Sensor for Real-Time Measurement of SF6 Decomposition Component H2S at the ppb Level. Anal. Chem..

[22] Sharma R.C., Kumar S., Gautam S., Gupta S., Srivastava H.B. (2017). Photoacoustic sensor for trace detection of post-blast explosive and hazardous molecules. Sens. Actuators B Chem..

[23] Zhang W., Wu Z., Yu Q. (2007). Photoacoustic spectroscopy for fast and sensitive ammonia detection. Chin. Opt. Lett..

[24] Alster M. (1972). Improved calculation of resonant frequencies of Helmholtz resonators. J. Sound Vib..

[25] Li Z., Liu J., Si G., Ning Z., Fang Y. (2022). Design of a high-sensitivity differential Helmholtz photoacoustic cell and its application in methane detection. Opt. Express.

[26] Zhao X., Chen K., Cui D., Guo M., Li C., Qi H., Zhang G., Gong Z., Zhou Z., Peng W. (2022). Ultrahigh sensitive photoacoustic gas detector based on differential multi-pass cell. Sens. Actuators B Chem..

[27] Zhang C., Qiao S., He Y., Zhou S., Qi L., Ma Y. (2023). Differential quartz-enhanced photoacoustic spectroscopy. Appl. Phys. Lett..

[28] Bonilla-Manrique O.E., Moser H., Martín-Mateos P., Lendl B., Ruiz-Llata M. (2019). Hydrogen sulfide detection in the midinfrared using a 3D-printed resonant gas cell. J. Sens..

[29] Dewey C.F., Kamm R.D., Hackett C.E. (1973). Acoustic amplifier for detection of atmospheric pollutants. Appl. Phys. Lett..

[30] Sampaolo A., Patimisco P., Giglio M., Zifarelli A., Wu H., Dong L., Spagnolo V. (2022). Quartz-enhanced photoacoustic spectroscopy for multi-gas detection: A review. Anal. Chim. Acta.

[31] Wu H., Dong L., Zheng H., Yu Y., Ma W., Zhang L., Yin W., Xiao L., Jia S., Tittel F.K. (2017). Beat frequency quartz-enhanced photoacoustic spectroscopy for fast and calibration-free continuous trace-gas monitoring. Nat. Commun..

[32] Guo M., Chen K., Yang B., Zhang G., Zhao X., Li C. (2022). Miniaturized anti-interference cantilever-enhanced fiber-optic photoacoustic methane sensor. Sens. Actuators B Chem..

[33] Chen K., Guo M., Yang B., Jin F., Wang G., Ma F., Li C., Zhang B., Deng H., Gong Z. (2021). Highly Sensitive Optical Fiber Photoacoustic Sensor for In Situ Detection of Dissolved Gas in Oil. IEEE Trans. Instrum. Meas..

[34] Chen K., Yu Z., Yu Q., Guo M., Zhao Z., Qu C., Gong Z., Yang Y. (2018). Fast demodulated white-light interferometry-based fiber-optic Fabry-Perot cantilever microphone. Opt. Lett..

[35] Lang Z., Qiao S., Ma Y. (2023). Fabry–Perot-based phase demodulation of heterodyne light-induced thermoelastic spectroscopy. Light Adv. Manuf..

[36] Zhou S., Slaman M., Iannuzzi D. (2017). Demonstration of a highly sensitive photoacoustic spectrometer based on a miniaturized all-optical detecting sensor. Opt. Express.

[37] Tan Y., Jin W., Yang F., Jiang Y., Ho H.L. (2019). Cavity-Enhanced Photothermal Gas Detection with a Hollow Fiber Fabry-Perot Absorption Cell. J. Light. Technol..

[38] Wang G., Fu D., Yuan S., Li C., Han X., Du J., Du F., Chen K. (2023). Rapid detection of dissolved acetylene in oil based on T-type photoacoustic cell. Microw. Opt. Technol. Lett..

[39] Li C., Guo M., Zhang B., Li C., Yang B., Chen K. (2022). Miniature single-fiber photoacoustic sensor for methane gas leakage detection. Opt. Lasers Eng..

[40] Kuusela T., Kauppinen J. (2007). Photoacoustic Gas Analysis Using Interferometric Cantilever Microphone. Appl. Spectrosc. Rev..

[41] Ushakov N., Lio L. (2014). Resolution limits of extrinsic Fabry-Perot interferometric displacement sensors utilizing wavelength scanning interrogation. Opt. Soc. Am..

